# Prevalence of *Plasmodium* parasites in non-human primates and mosquitoes in areas with different degrees of fragmentation in Colombia

**DOI:** 10.1186/s12936-019-2910-z

**Published:** 2019-08-19

**Authors:** Silvia Rondón, Cielo León, Andrés Link, Camila González

**Affiliations:** 1 Centro de Investigaciones en Microbiología y Parasitología Tropical, CIMPAT, Departamento de Ciencias Biológicas, Universidad de los Andes, Cra. 1 N° 18ª-12, Bogotá, Colombia; 2 Laboratorio de Ecología de Bosques Tropicales y Primatología, Departamento de Ciencias Biológicas, Universidad de Los Andes, Cra. 1 N° 18ª-12, Bogotá, Colombia; 3 Fundación Proyecto Primates, Cra. 11a N° 91-55, Apartamento 202, Bogotá, Colombia

**Keywords:** Fragmented forest, *Plasmodium*, *Anopheles*, Neotropical primates, Magdalena River valley

## Abstract

**Background:**

Parasites from the genus *Plasmodium,* the aetiological agent of malaria in humans, can also infect non-human primates (NHP), increasing the potential risk of zoonotic transmission with its associated global public health concerns. In Colombia, there are no recent studies on *Plasmodium* spp. infecting free-ranging NHP. Thus, this study aimed to determine the diversity of *Plasmodium* species circulating in fragmented forests in central Colombia, both in *Anopheles* mosquitoes and in the four sympatric NHP in the region (*Ateles hybridus, Cebus versicolor, Alouatta seniculus* and *Aotus griseimembra*), in order to evaluate the risk of infection to humans associated with the presence of sylvatic hosts and vectors infected with *Plasmodium* spp.

**Methods:**

Overall, there were collected 166 fecal samples and 25 blood samples from NHP, and 442 individuals of *Anopheles* spp. DNA extraction, nested PCR using mitochondrial (cox3 gene) and ribosomal (18S rDNA) primers, electrophoresis and sequencing were conducted in order to identify *Plasmodium* spp. from the samples.

**Results:**

*Plasmodium falciparum* was detected in two fecal samples of *Alouatta seniculus*, while *Plasmodium vivax/simium* infected *Ateles hybridus, Cebus versicolor* and *Alouatta seniculus*. Co-infections with *P. vivax/simium* and *Plasmodium malariae/brasilianum* were found in three individuals. The highest prevalence from blood samples was found for *Plasmodium malariae/brasilianum* in two *Alouatta seniculus* while *Plasmodium vivax/simium* was most prevalent in fecal samples, infecting four individuals of *Alouatta seniculus*. Seven *Anopheles* species were identified in the study site: *Anopheles (Anopheles) punctimacula, Anopheles (An.) malefactor, Anopheles (Nyssorhynchus) oswaldoi, Anopheles (Nys.) triannulatus, Anopheles (An.) neomaculipalpus*, *Anopheles (Nys.) braziliensis* and *Anopheles (Nys.) nuneztovari*. Infection with *P. vivax/simium* was found in *An. nuneztovari*, *An. neomaculipalpus*, and *An. triannulatus*. Furthermore, *An. oswaldoi* and *An. triannulatus* were found infected with *P. malariae/brasilianum*. The effect of fragmentation and distance to the nearest town measured in five forests with different degrees of fragmentation was not statistically significant on the prevalence of *Plasmodium* in NHP, but forest fragmentation did have an effect on the Minimum Infection Rate (MIR) in *Anopheles* mosquitoes.

**Conclusions:**

The presence of *Plasmodium* spp. in NHP and *Anopheles* spp. in fragmented forests in Colombia has important epidemiological implications in the human–NHP interface and the associated risk of malaria transmission.

## Background

Parasites belonging to the genus *Plasmodium* are among the best studied parasites in the world, since they are responsible of causing malaria, the deadliest vector borne disease [1]. Although malaria was targeted for elimination in 2030 in at least 35 countries, and the reduction of its incidence from 2005 until 2014 suggested this goal was achievable, in 2016 nine Latin American countries reported an increase in its incidence [2]. In Colombia, the number of cases increased in 2016, but most importantly, *Plasmodium falciparum,* the parasite responsible for cerebral malaria had an increase in its incidence, becoming more prevalent than *Plasmodium vivax*, the predominant species in the country until 2013 [2]. In 2018, the National System of Public Health Surveillance (SIVIGILA by its Spanish acronym) reported 61,339 cases of malaria in Colombia [3], *P. vivax* being the most prevalent (50%), followed by *P. falciparum* (48%), and mixed infection *P. vivax* and *P. falciparum* (2%).

Changes in malaria eco-epidemiology can be related to shifts in sylvatic transmission cycles, since *Plasmodium* parasites are also present in non-human primates (NHP) in tropical regions around the world [4]. Until now, 26 *Plasmodium* species have been formally described in NHP, each found infecting from one to 29 species [4].

In Latin America, NHP are potential reservoirs of *Plasmodium brasilianum/Plasmodium malariae* [5, 6], and some species have been found infected with *P. falciparum* [7] or with *Plasmodium simium* [5, 8], which is closely related to *P. vivax* [9, 10]. Although the risk of malaria zoonotic infection is of public health concern [11], it still remains largely understudied.

Several studies were carried out between 1930 and 1985 in Brazil, Panama, Venezuela, Peru and Colombia, and blood samples were obtained from the genera *Alouatta, Aotus, Cacajao, Callicebus, Callithrix, Brachyteles, Chiropotes, Lagothrix, Pithecia, Saimiri, Saguinus, Cebus, Callicebus* and *Ateles* [5, 8, 12–23]. They reported *P. simium* infecting *Alouatta guariba* and *Brachyteles arachnoides* [5, 8], and *P. brasilianum* infecting *Callicebus brunneus, Chiropotes satanas, Saguinus midas, Phitecia monachus, Lagothrix cana, Cebus capucinus, Saimiri sciureus, Saimiri boliviensis, Ateles geoffroyi* and *Alouatta palliata* [8, 16–18]. Most recent studies have been conducted in Brazil [24–29] and few others in Costa Rica [30] and Venezuela [6]. In Colombia, recent reports of *Plasmodium* infection on free-ranging primates are not available, and the last studies were conducted between 1952 and 1968. These early studies found evidence of *P. brasilianum* infecting NHP, including *Ateles geoffroyi, Cebus albifrons, Cebus apella, Cebus capucinus, Lagothrix lagotricha* and *Saimiri sciureus* [15, 17, 21].

The zoonotic risk of transmission between humans and NHP involves the presence of insect vectors feeding on both hosts; thus, mosquito feeding behaviour can influence *Plasmodium* transmission between humans and NHP [31]. In Africa, Makanga et al. documented that certain sylvatic mosquitoes infected with ape parasites also bite humans, being potential bridge vectors between humans and apes [32].

In Colombia, the most important malaria vectors are *Anopheles (Nyssorhynchus) albimanus, Anopheles (Nys.) darlingi* and *Anopheles (Nys.) nuneztovari* [33]. Studies on *Anopheles* mosquitoes in the country have been mainly focused in urban transmission cycles. However, in a context of pervasive transformation of natural areas into agricultural fields and extensive cattle ranches, it is essential to identify malaria vectors in forested areas, as these species may transmit *Plasmodium* from NHP to humans or vice versa [31].

In a socio-ecological context, the increasing human population coupled with greater demand for agricultural land, has led to an incremental deforestation in tropical countries where malaria is endemic [34]. It has been reported that deforestation and land use changes greatly influence malaria’s incidence [35]. In Colombia, the Middle Magdalena River valley is a region where the natural forest has been reduced to less than 15% of the original coverage due to deforestation and land use change [36]. This pervasive process might increase the contact between humans and NHP as well as affect parasite-host dynamics [37].

In this context, this study aimed to identify the prevalence of *Plasmodium* in five fragmented forest patches in Colombia in order to infer a potential risk of malaria zoonotic transmission involving NHP. The risk can exist if infected NHP species and infected *Anopheles* species are present in the study sites. To achieve this goal, the main objectives were: (i) to determine the presence and infection rate of *Plasmodium* parasites in NHP and *Anopheles*, (ii) to establish if infection rates vary among infected *Anopheles* species, and (iii) to evaluate if sites with different degrees of habitat transformation exhibit variation in infection rates.

## Methods

### Study sites

**Fig. 1:**
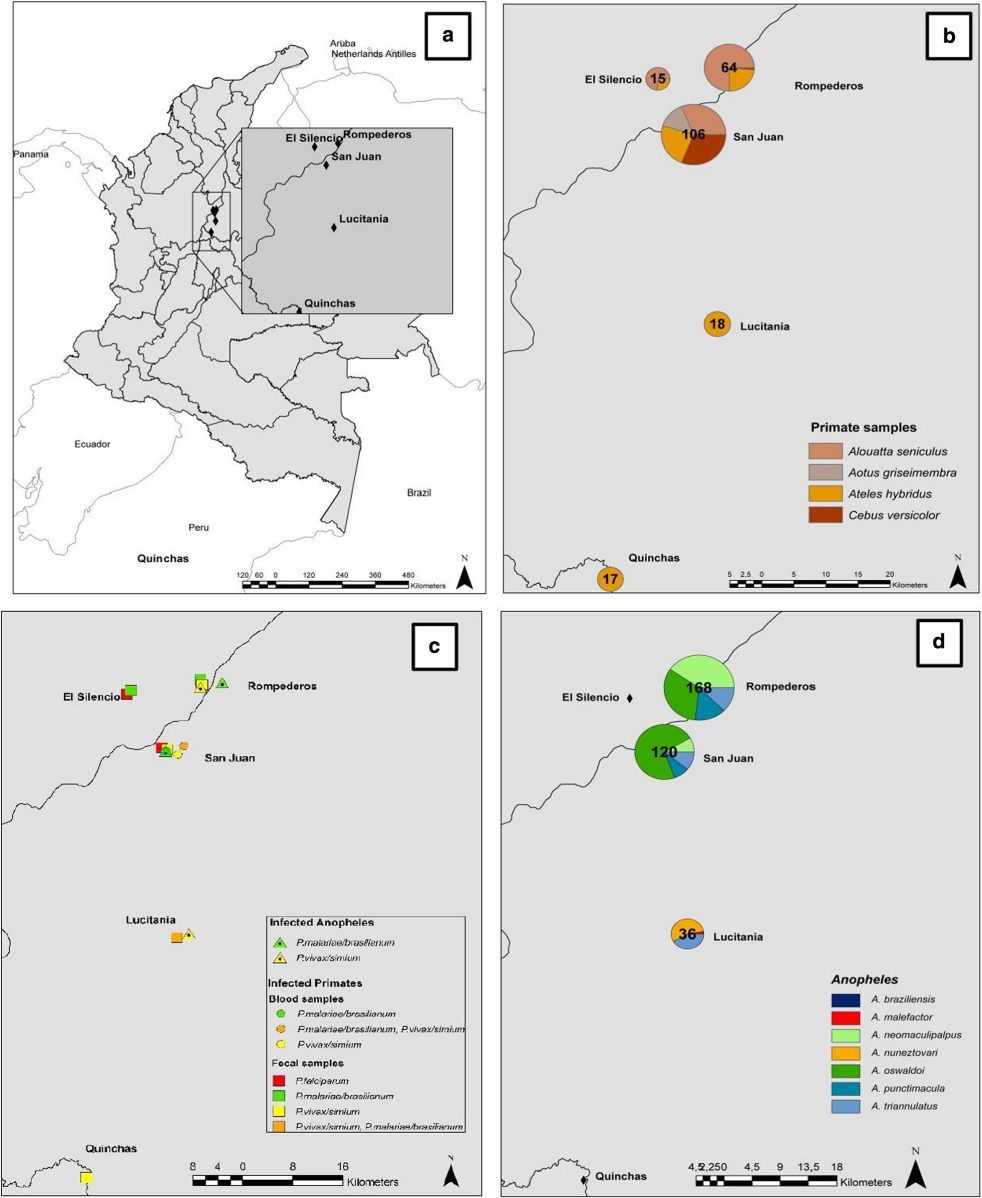
**a** Study sites. **b** Number of NHP samples per species and study site. **c** *Plasmodium* infection presence in *Anopheles* and NHP samples (feces and blood) per study site. **d** *Anopheles* species collected per study site

Fieldwork was performed in five forest fragments (San Juan, Lucitania, Rompederos, El Silencio, and Quinchas) located in the Middle Magdalena River valley in Santander, Antioquia and Boyaca Departments, in Colombia (Table 1 and Fig. 1a). In the Department of Santander malaria transmission occurs mainly in two municipalities, Cimitarra and Puerto Wilches. In 2018, *P. vivax* was the dominant species [38] with 22 cases, while seven were notified of *P. falciparum* and one co-infection [3]. In the Department of Antioquia in 2018, *P. vivax* was detected in 4360 of the notified cases, *P. falciparum* in 975 and 80 co-infections [3]. In the Department of Boyacá fewer cases are known, with only four records in 2017 in the municipality of Puerto Boyacá [39], and none in 2018 [40].

**Table 1:** Study sites information and sampling dates

Study site	Coordinates	Department	Forest fragment size (ha)	Distance to nearest town (m)	Fecal samples collection	Blood samples collection	Entomological collection
San Juan	06°43′ N 74°09′ W	Santander	65	8232.1	June 2016, September–December 2017, January 2018	March–September–December 2017, January 2018	December 2017
Lucitania	06°26′ N 74°07′ W	Santander	13	16,776.1	August 2017		January 2018
Rompederos	06°49′ N 74°06′ W	Antioquia	36	4979.4	June 2017, January 2018		January 2018
El Silencio	06°48′ N 74°12′ W	Antioquia	30	8391.9	June 2017		
Quinchas	06°03′ N 74°16′ W	Boyacá	250	5701.7	January 2013

### Non-human primate samples

Between 2016 and 2018, primates were followed from dawn to dusk and 166 fecal samples were collected from the soil immediately after defecation, and placed in eppendorf tubes with RNA*later* solution. Fecal samples from brown spider monkeys (*Ateles hybridus),* capuchin monkeys (*Cebus versicolor*), red howler monkeys (*Alouatta seniculus*) and grey-legged night monkeys (*Aotus griseimembra*) were collected (Table 2 and Fig. 1b). Additionally, in San Juan, blood samples were collected from 25 primates (Table 2) anesthetized using darts with zolazepam hydrochloride (Zoletil), with specific doses for each species. Once sedated, blood samples were collected by caudal vein puncture, and placed in Vacutainer tubes containing sodium citrate. Plasma and red blood cells were separated by centrifugation and stored in liquid nitrogen tank (Thermo Scientific) until transported to the laboratory. For 16 specimens (three *Alouatta seniculus*, six *Aotus griseimembra*, two *Ateles hybridu*s and five *Cebus versicolor*) blood and fecal samples were obtained from the same individuals in the same field campaign.

**Table 2:** Number of samples per study site and primate species

Study site	Primate species	Fecal samples	Blood samples
San Juan	*Alouatta seniculus*	23	5
	*Cebus versicolor*	24	9
	*Ateles hybridus*	21	4
	*Aotus griseimembra*	8	7
Lucitania	*Ateles hybridus*	18	
Rompederos	*Alouatta seniculus*	24	
	*Cebus versicolor*	1	
	*Ateles hybridus*	15	
El Silencio	*Alouatta seniculus*	11	
	*Ateles hybridus*	4	
Quinchas	*Ateles hybridus*	17	
Total		166	25

### Mosquito collection and identification

Adult mosquitoes were sampled in three study sites (Rompederos, San Juan, and Lucitania) during three consecutive nights in each site; due to transport and accessibility restrictions, the remaining two sites were not sampled for mosquitoes (Quinchas and El Silencio). One Shannon trap [41, 42], three BG-Sentinel traps (BioGents, Regensburg, Germany) [43] baited with Octenol and six CDC light-traps [44, 45] (three on the canopy and three on the understorey) were set, close to places frequented by primates.

Mosquitoes were sorted immediately after capture and female *Anopheles* were preserved in RNA*later* buffer. Some females were kept dry for morphologic identification using the keys of González and Carrejo [46] and Forattini [47]. Species identity was confirmed through DNA barcoding with amplification of the 658 bp region from the COI gene [48, 49].

### Molecular analyses

DNA from fecal samples was extracted using a ZR fecal DNA MiniPrep Kit (Zymo), according to the manufacturer's protocol. DNA extraction from NHP blood samples was performed individually using High Pure PCR Template Preparation Kit (Roche). Female mosquitoes were pooled by species and study site, and DNA was extracted from pools with up to ten individuals using the ZR Tissue & Insect DNA MiniPrep Kit (Zymo).

To detect *P. falciparum*, *P. vivax/simium* and *P. malariae/brasilianum*, nested PCR using mitochondrial primers (cox3 gene) and nested PCR using ribosomal primers (18S rDNA) were performed in all samples following published methods [50, 51]. All PCR products from second reactions were visualized on an agarose gel and positive samples were sequenced by Big Dye Terminator v3.0 Cycle Sequencing Kit using the ABI-3500 Genetic Analyzer (Life Technologies) for species identity confirmation. Sequences were edited using *Geneious* Software and compared by BLAST (Basic Local Alignment Search Tool) [52] with publicly available sequences in GenBank (National Center for Biotechnology Information).

### Data analyses

The prevalence (infection rate) of *Plasmodium* spp. in each NHP species and study site was calculated as # infected individuals/total examined individuals * 100. The minimum infection rate (MIR) in each *Anopheles* species was calculated as the number of positive pools divided by the total of tested specimens, assuming that each positive pool contains at least one infected individual [49].

A General Linear Model (Family: quasibinomial) was performed using the RStudio integrated development environment, in order to evaluate the effect of fragmentation and distance to the nearest town on *Plasmodium* prevalence in NHP and the MIR. Forest fragment size (ha) was used as a proxy for fragmentation intensity [53].

## Results

### Non-human primate samples

*Plasmodium* spp. infection was detected in 20 NHP samples. *Plasmodium falciparum* was found infecting *Alouatta seniculus* (n = 2), while *P. vivax/simium* infected *Ateles hybridus* (n = 5), *Cebus versicolor* (n = 2) and *Alouatta seniculus* (n = 5). Additionally, *P. malariae/brasilianum* was found infecting the four NHP sampled species (Table 3 and Fig. 1c). Co-infections with *P. vivax/simium* and *P. malariae/brasilianum* were found in two individuals of *Ateles hybridus* in Lucitania and one individual of *Alouatta seniculus* in San Juan. For those specimens with paired fecal and blood samples, consensus in infection results was not obtained since five individuals were only positive for fecal samples and seven for blood samples.

**Table 3:** Prevalence (%) of *Plasmodium* spp. and number of positive samples per study site and primate species

Study site	Primate species	Fecal samples			Blood samples		
		*P. falciparum*	*P. vivax/simium*	*P. malariae/brasilianum*	*P. falciparum*	*P. vivax/simium*	*P. malariae/brasilianum*
San Juan	*Alouatta seniculus*	1 (4.3%)				1 (20%)^a^	2 (40%)^a^
	*Cebus versicolor*		2 (8.3%)				1 (11.1%)
	*Ateles hybridus*					1 (25%)	1 (25%)
	*Aotus griseimembra*			1 (12.5%)			1 (14.2%)
Lucitania	*Ateles hybridus*		2 (11.11%)^a^	2 (11.11%)^a^			
Rompederos	*Alouatta seniculus*		4 (16.6%)	1 (4.1%)			
	*Cebus versicolor*						
	*Ateles hybridus*		1 (6.6%)				
El Silencio	*Alouatta seniculus*	1 (9.0%)					
	*Ateles hybridus*						
Quinchas	*Ateles hybridus*		1 (5.8%)				

^a^Co-infections with *P. vivax/simium* and *P. malariae/brasilianum*. For specimens with paired blood and fecal samples, consensus in infection results was not obtained

The higher *Plasmodium* prevalence was obtained with *P. malariae/brasilianum* in *Alouatta seniculus* blood samples and *Ateles hybridus* fecal samples (Table 3).

*Plasmodium* prevalence in NHP was not related to fragmentation (General linear model: fecal samples Pr = 0.415, blood samples Pr = 0.272) or distance to nearest town (General linear model: fecal samples Pr = 0.272, blood samples Pr = 0.272).

### Mosquito collection

In total, 442 mosquitoes were collected, but due to loss of important taxonomic characters during processing and transport, 26.6% of them could not be identified and were not processed for parasite infection. The remaining 324 mosquitoes were collected mainly in Rompederos (51.8%), followed by San Juan (36.7%) and Lucitania (10.8%).

In this study, the Shannon trap was the most efficient capture method with 430 *Anopheles.* CDC light-traps caught 12 *Anopheles* (only one from the canopy) while BG-Sentinel traps did not capture any *Anopheles.* Seven *Anopheles* species were identified based on diagnostic morphological characters: *Anopheles (Anopheles) punctimacula, Anopheles (An.) malefactor, Anopheles (Nys.) oswaldoi, Anopheles (Nys.) triannulatus, Anopheles (An.) neomaculipalpus*, *Anopheles (Nys.) braziliensis* and *Anopheles (Nys.) nuneztovari* (Fig. 1d). The species identity of *An. punctimacula, An. malefactor, An. oswaldoi, An. triannulatus,* and *An. neomaculipalpus* was confirmed by DNA barcoding, obtaining 99–100% of identity with the reference sequences in GenBank. The remaining two species *An. braziliensis* and *An. nuneztovari* did not have successful amplification of the COI gene, and the obtained sequences could not be validated, but morphologic identification was successfully performed based on diagnostic characters. *Anopheles triannulatus* was found in all three sampling sites, and *An. oswaldoi* and *An. neomaculipalpus* were the most abundant species (Table 4).

**Table 4:** Results of infection in *Anopheles* per site, showing the number of *Anopheles* tested, number of infected pools, minimum infection rate (MIR) and number of positive pools by *Plasmodium* species

Study site	Species	Total Anopheles tested	Infected pools	MIR	*P. vivax*	*P. malariae*	*P. falciparum*
Rompederos	*An. punctimacula*	24	0	0	0	0	0
	*An. neomaculipalpus*	68	3	0.04	3	0	0
	*An. triannulatus*	21	1	0.05	0	1	0
	*An. oswaldoi*	55	1	0.02	0	1	0
	Total	168	5				
Lucitania	*An. triannulatus*	15	1	0.07	1	0	0
	*An. nuneztovari*	20	2	0.1	2	0	0
	Total	35	3				
San Juan	*An. oswaldoi*	86	1	0.01	0	1	0
	*An. punctimacula*	10	0	0	0	0	0
	*An. neomaculipalpus*	10	0	0	0	0	0
	*An. triannulatus*	13	0	0	0	0	0
	*Total*	119	1				
Total		322	9				

Infection with *P. vivax/simium* was found in *An. nuneztovari, An. neomaculipalpus*, and *An. triannulatus.* Furthermore, *An. oswaldoi* and *An. triannulatus* were found infected with *P. malariae/brasilianum* (Table 4). The MIR was related to fragmentation (General linear model: Pr = 0.002), but not to proximity to the nearest town (General linear model: Pr = 0.056).

## Discussion

In this study, three *Plasmodium* species were found infecting NHP in the Magdalena River valley. As expected, the most prevalent parasite species was *P. malariae/P. brasilianum* that naturally infects different NHP species in Central and South America [6, 25, 30]. Interestingly, *P. brasilianum* has been reported infecting humans living in close proximity with NHP in the Venezuelan Amazon [6], which highlights the risk of parasite transmission from NHP to humans.

The finding of *P. vivax/P. simium* in NHP is of great interest given that *P. vivax* has been the most prevalent species historically recorded in the country. In 2017, after analysing 28 human blood samples from an outbreak in the Atlantic Forest coastal region of Brazil, *P. simium* was detected infecting humans [24]. Authors suggested that this species could be circulating in humans before, but was misdiagnosed as *P. vivax* due to the absence of adequate diagnostic techniques to perform species identification. Additionally, Grigg and Snounou (2017) consider Brazilian monkeys as reservoirs for *P. vivax* [54]. The presence of sylvatic reservoirs is relevant as it can potentially threaten successful malaria elimination campaigns [54]. In the Colombian context the presence of infected monkeys should be taken into account when elucidating the potential risk of human infection.

In our study, the unexpected presence of *P. falciparum* infecting *Alouatta seniculus* open very relevant questions and concerns. Although this species has been found in New World primates [7, 55] it is not as prevalent as *P. brasilianum*. The presence of *P. falciparum* in NHP suggests parasite transmission from humans to NHP, which is relevant when evaluating human contribution to emerging infectious diseases in sylvatic NHP. Other vector borne diseases transmitted from humans to primates such as Yellow Fever in South America, have negatively impacted populations of *Alouatta guariba clamitans* and *Alouatta caraya* in Argentina and Brazil, reassessing their conservation status to Critically Endangered and Near Threatened respectively [56]. Regarding the establishment of *P. falciparum* in the wild, Araújo et al. considered that, due to the ability of the parasite to develop in NHP, mosquito infection from infected NHP is likely and so is the establishment of a sylvatic transmission cycle [7].

Although infection with *Plasmodium* species has been reported in the genera *Alouatta, Cebus, Aotus* and *Ateles* [7, 8, 17], results obtained in this study constitute new records at the species level for *Cebus versicolor, Ateles hybridus* and *Aotus griseimembra*.

Fecal samples have been used in different studies mainly in Africa and Asia [57, 58] for *Plasmodium* detection as a simple, non-invasive and inexpensive alternative to blood samples. However, they degrade quickly [58] and the presence of bacteria and polysaccharides from plant diet, which are potential inhibitors of PCR, makes it difficult to use these samples for diagnosis by PCR [59]. To solve this inconveniences, there was used RNA*later* for sample preservation and BSA in the PCR mix in order to stabilize the DNA [60]. Since *Plasmodium* detection from blood is more sensitive than from fecal samples [57, 61], prevalence rates found in this study may be underestimated, given that blood samples could not be obtained for all sampled primates. Also, those differences in detection sensitivity according to the type of sample could partially explain the lack of coincidence in the obtained results for the tested paired fecal and blood samples. For future studies, in order to confirm *Plasmodium* species circulating in zoonotic cycles, whole genome sequencing should be performed. In this way, it is possible to determine if *Plasmodium* infections are caused by *P. simium* or *P. vivax*, and *P. malariae* or *P. brasilianum.*

Regarding mosquito collections, all the species found in this study were known records for the study sites: *An. nuneztovari, An. triannulatus, An. neomaculipalpus* and *An. oswaldoi* have been previously reported in Santander Department [62, 63], and *An. punctimacula, An. neomaculipalpus* and *An. triannulatus* in Antioquia Department [33], as well as *An. oswaldoi* [64].

*Plasmodium vivax* was found infecting three species, *An. neomaculipalpus, An. triannulatus* and *An. nuneztovari,* which is concordant with previous records [65, 66]. *Anopheles neomaculipalpus* is known to be highly anthropophilic [65] while *An. triannulatus* has been collected resting on cattle and is known to colonize transformed environments and become very abundant [67]. Lucitania, Rompederos and San Juan exhibit the presence of cattle and altered ecosystems mainly due to the progressive introduction of oil palm plantations. It has been reported that deforestation related to monoculture favours the presence of ponds which are frequent breeding sites of *An. nuneztovari* [68], a species recognized as primary malaria vector in Colombia [69].

Infection with *P. malariae* was found in *An. triannulatus* and *An. oswaldoi* as has also been previously recorded [70, 71]. *Anopheles triannulatus* has been reported with zoophilic and anthropophilic activities [33] and *An. oswaldoi* has been incriminated as secondary vector in the country [69]. *Anopheles triannulatus* and *An. oswaldoi* were found in this study, supporting previous reports and confirming their presence in Santander and Antioquia Departments.

The most abundant species were *An. oswaldoi* and *An. neomaculipalpus* found infected with *P. vivax* and *P. malariae,* respectively; this suggests their potential role as vectors in the study sites. Rompederos and Lucitania were the localities with highest MIR. *Anopheles* vectors and NHP exhibit different selection strategies; while the lifespan of NHP is long, probably supporting a long course of infection, mosquitoes have a short lifespan and marked fluctuation in population densities related to environmental variables [72, 73]. Possibly the sampling time of this study coincided with low *Anopheles* local abundances thus the number of collected individuals was below the detection threshold. Long-term studies including seasonal variation would allow a better understanding of hosts’ population dynamics and the parasite transmission system in the studied environment.

Regarding the diversity of *Anopheles* species it was higher in Rompederos and San Juan, compared to Lucitania. This could be explained partially by the fact that those fragments belong to flooded forests which could provide optimal mosquito breeding sites, while Lucitania is a terra firme fragment forest.

The analyses on the effect of habitat fragmentation and distance to nearest town over the prevalence of *Plasmodium* in NHP didn’t show any significant association, while the fragmentation but not the distance to nearest town had an effect over the MIR. However, when evaluating malaria risk, it is important to consider that multiple factors and processes interact e.g. the environment (land cover use/change, landscape transformations), human populations (host susceptibility, movement patterns, forest-related activity), vector biology (vector activity and life cycle, mosquito species distribution) [74]. This study only focused in some of those factors (e.g.: *Plasmodium* infection rates), but the complexity of the malaria cycle is a fact that must be taken into account, and for further studies it is suggested to involve as many factors as possible.

## Conclusions

The results of this study provide evidence for a potential risk of zoonotic malaria transmission in terms of *Plasmodium* species infecting NHP and *Anopheles,* which can have negative effect on both human and NHP populations. Fragmentation and proximity to the nearest town did not show a statistically significant effect on the prevalence of *Plasmodium* in NHP, while fragmentation had an effect over the MIR.

## Data Availability

All data generated during this study are included in this published article.

## Abbreviations

BLAST: Basic Local Alignment Search Tool
COI: cytochrome c oxidase subunit I
DNA: deoxyribonucleic acid
MIR: minimum infection rate
NHP: non-human primates
PCR: polymerase chain reaction
